# Laproscopic treatment for small bowel bleeding after detection by double-balloon endoscopy: A case report

**DOI:** 10.1016/j.ijscr.2019.05.003

**Published:** 2019-05-10

**Authors:** Shintaro Akabane, Takahisa Suzuki, Takao Hinoi, Yosuke Shimizu, Takeshi Sudo, Takashi Onoe, Kohei Ishiyama, Wataru Shimizu, Hirofumi Tazawa, Naoto Hadano, Toshihiro Misumi, Masato Kojima, Haruna Kubota, Junichi Zaitsu, Daiki Taniyama, Kazuya Kuraoka, Hirotaka Tashiro

**Affiliations:** a Department of Surgery, National Hospital Organization, Kure Medical Center and Chugoku Cancer Center, 3-1, Aoyama-cho, Kure city, Hiroshima, 737-0023, Japan; b Department of Pathology, National Hospital Organization, Kure Medical Center and Chugoku Cancer Center, 3-1, Aoyama-cho, Kure city, Hiroshima, 737-0023, Japan; c Department of Gastroenterological and Transplant Surgery, Applied Life Sciences, Institute of Biomedical & Health Sciences, Hiroshima University, 1-2-3, Kasumi, Minami-ku, Hiroshima, 734-8551, Japan

**Keywords:** AVM, arteriovenous malformation, DBE, double-balloon endoscopy, Arteriovenous malformation (AVM), Double-Balloon endoscopy, Laparoscopic small bowel resection

## Abstract

**Introduction:**

The frequency of small bowel bleeding is relatively low and the process for the diagnosis and treaVtment remains difficult. Here, we report a case of massive small bowel bleeding due to arteriovenous malformation (AVM), treated by a combination of double-balloon endoscopy and laparoscopic resection.

**Presentation of Case:**

A 59-year-old man was admitted to our hospital due to a hemorrhagic stool. The patient presented transient hemorrhagic shock and contrast-enhanced CT revealed a hyper-vascularized tumor in the small bowel. India ink tattooing for the responsible lesion with double-balloon endoscopy was performed. The tattooed lesion was easily confirmed during the subsequent laparoscopic observation and segmental resection was done. Pathological examination showed arteriovenous malformation of the small bowel.

**Discussion:**

Prior to laparoscopic resection, the localization of the responsible area might be a significant consideration when the lesion is invisible. Endoscopic marking with DBE enables intraluminal detection and laparoscopic observation from the serosal side.

**Conclusion:**

Preoperative marking with the use of double-balloon endoscopy followed by laparoscopic resection might be an optimal option for the treatment of massive small intestinal bleeding.

## Highlights

• The frequency of small bowel bleeding due to an arteriovenous malformation is rare.
• Localization of the bleeding location is required prior to laparoscopic resection.
• Double-balloon endoscopy enabled direct observation and preoperative tattooing.
• Endoscopic marking followed by laparoscopic resection might be optimal option.

## 1 Introduction

Small bowel disease is relatively rare and the procedure for diagnosis and treatment can be challenging [1]. Although selective mesenteric angiography [2] and video capsule endoscopy [3] have been reported as useful diagnostic tools, double-balloon endoscopy (DBE) has been recognized as a promising diagnostic device because it provides subsequent treatment options [3]. Localization of the responsible lesion is required prior to the laparoscopic resection when it is invisible or impalpable from the serosal side. We herein present a rare case of acute small intestinal bleeding caused by arteriovenous malformation (AVM) localized by DBE and followed by curative resection in laparoscopic surgery. This work has been reported in line with the SCARE criteria [4].

## 2 Presentation of case

**Fig. 1:**
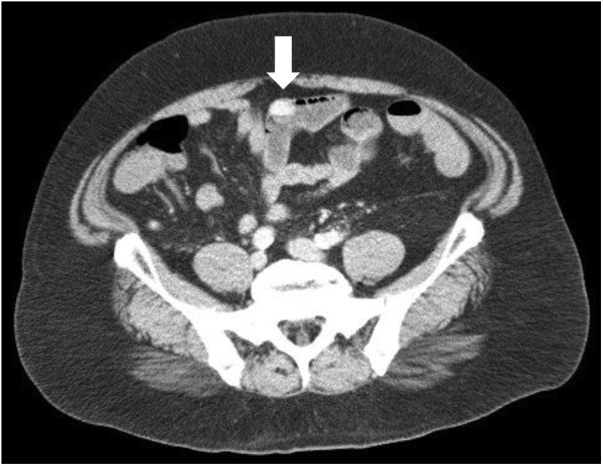
Contrast-enhanced CT shows a hyper-vascularized tumor in the small bowel (white arrow).

**Fig. 2:**
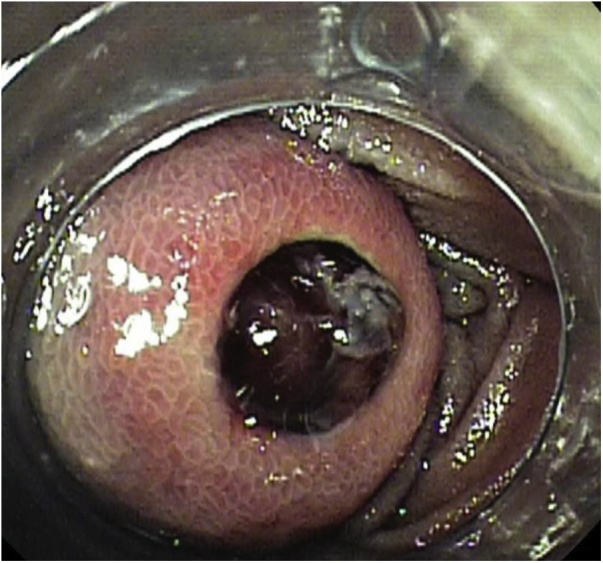
Double-balloon endoscopy revealed an ulcerated lesion and massive bleeding from an exposed vessel.

A 59-year-old man was admitted to the emergency room complaining of intermittent melena for three days. The patient had a body mass index of 38.3 kg/m^2^ with a history of hypertension, type 2 diabetes mellitus, liver cirrhosis, esophagus varices and hyperthyroidism. Complete blood count showed severe anemia with a hemoglobin level of 8.9 g/dL. The patient went into hemorrhagic shock, with a systolic blood pressure of 70 mmHg. Four units of blood infusion was performed and vital signs were recovered. Contrast enhanced CT showed a hyper-vascularized tumor in the small bowel which was considered to be a bleeding location (Fig. 1). Subsequently, double-balloon endoscopy with an oral approach was performed and a submucosal tumor with ulcerated lesion was identified (Fig. 2). Massive bleeding from an exposed vessel in the ulcerative location was confirmed and India ink tattooing combined with clipping the adjacent mucosa was done for a subsequent laparoscopic resection. A preoperative X-ray image showed the clipped lesion in the right-upper abdomen.

**Fig. 3:**
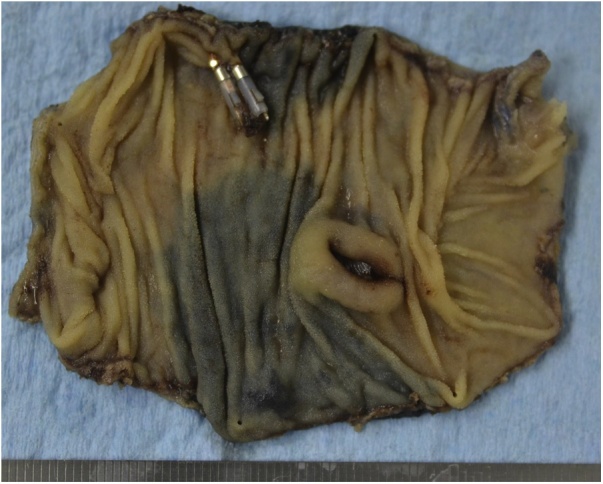
Resected specimen showing submucosal tumor with ulcerated lesion.

**Fig. 4:**
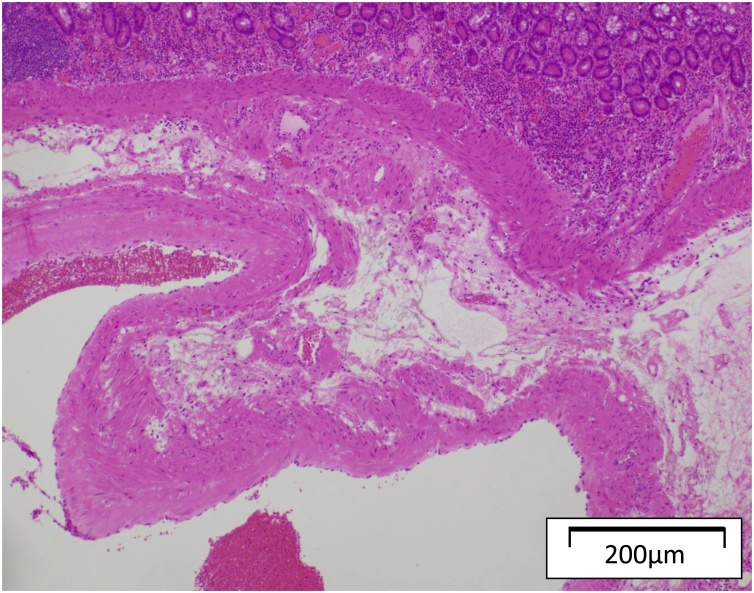
Significantly dilated vascular region in the submucosal layer and a mucosal inversion into the disrupted vessel were confirmed by histological examination.

The patient was sent to the operation room and laparoscopic surgery was initiated in a supine position with general anesthesia. An umbilicus incision with a diameter of 20 mm was placed and a 10-mm flexible scope was inserted under 10 mmHg of abdominal air pressure. Two small incisions (5 mm) were made in the left-upper and right-lower abdomen taking into account the tumor location speculated from the preoperative X-ray image. The tattooed location in the small intestine was identified and grabbed with non-crushing forceps. The umbilicus incision was extended to 40 mm and was protected by the wound retractor. The marked lesion was extracted to outside of the peritoneal cavity 24 min after the start of operation and partial resection with functional end-to-end anastomosis was performed. A submucosal tumor with a diameter of 14 mm x 10 mm was detected in the resected specimen, which was responsible for the massive bleeding (Fig. 3). Histological examination revealed a significantly dilated vascular region in the submucosal layer and inversion of mucosa was detected at the disrupted location of the vessel (Fig. 4). Pathological diagnosis provided a conclusion of arteriovenous malformation. The patient was discharged on the 7th postoperative day and the postoperative course was uneventful to date.

## 3 Discussion

The occurrence of acute small intestinal bleeding is comparably rare and accounts for approximately 5% of all acute gastrointestinal bleeding [5]. Small bowel bleeding is generally caused by tumors, ulcerated lesions, diverticulums and vascular malformations [6]. AVM is characterized by a thin vessel with a defect in smooth muscle [7] and massive bleeding caused by AVM can be fatal [1]. Therefore, prompt diagnosis followed by appropriate treatment is required.

Previous studies have shown that identification of small bowel bleeding is very difficult because of its inaccessibility with conventional endoscopy [6]. Selective mesenteric angiography and embolization might be one option as a diagnostic tool or a bleeding control strategy, however, it has a risk of extensive intestinal ischemia. Recently, video capsule endoscopy has been reported as a minimally invasive diagnostic tool to confirm obscure gastrointestinal hemorrhage [3]. Moreover, DBE allows direct observation and is preferentially recommended in emergency cases, especially for massive bleeding, because it provides treatment options, such as coagulation or endoscopic marking [3].

A previous study conducted by Richter et al. showed that surgical resection of the angiodysplasia had a greater reduction rate for recurrent bleeding in the lower digestive tract than medical or endoscopic treatment [8]. As for surgical intervention, laparoscopic surgery in small bowel resection has more advantages, such as a reduction of adhesions and incisional hernias compared to laparotomy. In this case, laparoscopic investigation following endoscopic marking helped in prompt identification of the responsible lesion. Siu et al. reported in their randomized controlled trial of emergency surgery for perforated peptic ulcer that laparoscopic surgery resulted in less postoperative pain, decreased chest complication and shorter hospitalization than open surgery [9]. This patient was at risk of perioperative complication due to severe obesity with a BMI > 35, therefore, laparoscopic resection was even more meaningful in promoting postoperative recovery.

Prior to laparoscopic resection, the localization of the responsible area might be a significant consideration when the lesion is invisible. There have been some reports about localizing procedures following selective mesenteric angiography, such as methylene blue injection [10], placement of metallic coil [2], and intraoperative indocyanine green injection via the microcatheter and fluorescent scope observation [11]. Unlike these procedures, endoscopic marking with DBE enables intraluminal detection and laparoscopic observation from the serosal side without an X-ray device [6]. In this case, India ink tattooing with DBE made it possible to detect the localized lesion and to resect a minimum length of the small bowel.

## 4 Conclusion

This case report supports the efficacy of preoperative marking with the use of DBE followed by laparoscopic resection in the treatment of massive small intestinal bleeding.

## Conflicts of interest

None of the authors has anything to disclose.

## Funding

None of the authors has anything to disclose

## Ethical approval

All procedures used in this research were approved by the Ethical Committee of Kure Medical Center and Chugoku Cancer Center.

Consent

Written informed consent was obtained from the patient for publication of this case report and any accompanying images. A copy of the written consent is available for review by the Editor-in-Chief of this journal.

## Author contributions

All of the authors read and approved the final manuscript. Authors’ contributions are as follows; SA, TS, and HT were responsible for the conception and design of this study. SA wrote the manuscript and literature search. TS and HT reviewed and edited the manuscript. SA, TS, TH, YS, WS, TS, TO, KI, HT, NH, TM, MK, HK and HT treated and observed the patient. JZ, DT and KK performed the pathological analysis.

## Guarantor

Takahisa Suzuki has accepted full responsibility for this work and the decision to publish it.

## Provenance and peer review

Not commissioned, externally peer-reviewed
